# Supplementary material for: Berberine Effects in Pre-Fibrotic Stages of Non-Alcoholic Fatty Liver Disease—Clinical and Pre-Clinical Overview and Systematic Review of the Literature
Source: Int J Mol Sci. 2024 Apr 10;25(8):4201. doi: 10.3390/ijms25084201 (PMC11050387; doi:10.3390/ijms25084201)
Supplement: Supplementary file 1 [file ijms-25-04201-s001.zip › ijms-2935152-supplementary.pdf]

Supplementary Table S1 – A detailed overview of Berberine and its effects in preclinical studies.

Authors	Experiment and model type	Pharmaceutical intervention and dosage	Subject	Results and key impacts on NAFLD	Pathway
Sharma A et al. (2020) [141]	Cell cultures HepG2 and Hepa 1-6<br>In vitro	Berbamine (BBM) – different concentrations of (1, 2 and 5 μM)		Activation of AMP activated kinase and peroxisome proliferator-activated receptor PPAR – α downregulated SREBP-1c expression, thus BBR was able to modulate stress oxidative response ($p<0.001$) and lipid metabolism by preventing the accumulation of fat droplets in cells.	**AMPK/mTOR/SREBP-1c and AMPK/Nrf2 modulate lipid metabolism, inflammatory and oxidative stress.**<br>**Western blot for key proteins and enzymes**
Zhu X. et al. (2019) [127]	C57BL/6 J and ob/ob mice<br>HepG2 and AML12 cells<br>In vivo and in vitro<br><br>And 6 liver biopsies from NAFLD patients	BBR (300 mg/kg/day) – in vivo<br><br>BBR (20 μM) – in vitro	Not mentioned	BBR treatment was able to downregulate the expressions of SCD1 and SREPBP-1c both in vivo and vitro. By doing so BBR managed to exert an anti-steatotic effect.<br>**Secondary endpoints**:<br>BBR treatment reduced hepatic lipid synthesis and prevented TG accumulation in liver both in animal model and cell cultures.	**AMPK-SREBP-1c-SCD1 pathway**<br>**Western blot for key proteins and enzymes**<br>**Liver enzyme profile and serum lipid profile.**<br>**Histological analysis**
Zhang YP. et al. (2019) [97]	Rats Sprague-Dawley<br>In vivo	BBR 100 mg/kg intragastrically once a day (16 weeks)	48 (16/16/16)	By activating SIRT3/AMPK/ACC pathway, BBR exerted anti-steatotic effects and improved lipid metabolism in steatotic induced animal model.<br>**Secondary endpoints**:	**SIRT3/AMPK/ACC pathway**<br>**Liver enzyme profile and serum lipid profile**<br>**Western blot for key proteins and enzymes**

Supplementary Table S1 – A detailed overview of Berberine and its effects in preclinical studies.

		(1) control group<br>(2) HFD<br>(3) BBR		Histologically BBR treatment resulted in an improvement of hepatic steatosis. BBR had hepatoprotective action and can ameliorate lipid accumulation.	**Histological analysis**
Li QP. et al. (2021) [89]	Sprague Dawley rats<br>In vivo	Oxyberberine (OBB)<br>(8 weeks)<br>(1) control group<br>(2) HFD group)<br>(3) Met 300 group (300 mg/ kg metformin)<br>(4) OBB-25 group (25 mg/kg OBB)<br>(5) OBB-50 group (50 mg/ kg OBB)<br>(6) OBB-100 group (100 mg/kg OBB),	Not mentio ned	OBB an oxidative metabolite of BBR had superior effect on activation of AMPK pathway in liver and adipose tissue ($p<0.05$), therefor it had stronger hepatoprotective properties compared to BBR in steatotic induced animal model.<br>**Secondary endpoints**:<br>Improvement in liver enzymes and glucose metabolism compared to BBR.<br>OBB (100 mg/kg) had superior hepatoprotective effects (lower AST and ALT levels) and had better results regarding lowering lipid content than BBR. (100 mg/kg) - $p<0.05$.<br>OBB exerted anti-inflammatory properties as shown by a decrease in TNF-α levels.	**Histological analysis**<br>**Liver enzymes profile and serum lipid profile**<br>**RT-PCR and Western blot for key proteins and enzymes.**<br>**Activation of AMPK pathway**

Supplementary Table S1 – A detailed overview of Berberine and its effects in preclinical studies.

		(7) BRR-100 group (100 mg/ kg).			
Choi YJ. et al. (2017) [131]	MICE C57BL/6<br>CELL CULTURES HepG2<br><br>In vivo and in vitro	BBR (**10 mg/kg/day; i.p.)** – intraperitoneal<br><br>(1) control group<br>(2) three BBR receiving groups		BBR treatment promoted steatosis both in vivo and in vitro by activating AMPK pathway, thus increasing CD36 levels via ERK-C/EBPβ pathway.<br>**Secondary endpoints**:<br>Lipid serum profile was elevated after BBR treatment ($p<0.05$)<br>BBR treatment altered liver enzymes profile as shown by an increase in both AST and ALT.	**Liver enzyme profile and serum lipid profile**<br>**Histological analysis**<br>**Western blot for key proteins and enzymes**<br>**Immunofluorescence microscopy**<br>**Activation of AMPK, ERK-C/EBPβ pathways and CD36 expression.**
Qiang X. et al. (2016) [133]	MICE<br>C57BLKS/J (db/db)<br>ICR mice<br>In vivo and in vitro	Demethyleneberberine (DMB)<br><br>In vivo:<br>N=32<br>(1) control group<br>(2) the methionine and choline deficient (MCD) group	32(8/8/8/8)<br>8(4/4)	DMB works by activating AMPK pathway and by increasing ACC levels both in vivo and vitro, as well as upregulating fatty acid oxidation.<br>**Secondary endpoints**:<br>Improvement in liver and serum lipid profile<br>Moreover, DMB was able to reduce the hepatic oxidative and inflammatory state, resulting in protecting livers from steatosis-related injuries.<br>DMB treatment was associated with histological improvement.	**Liver enzymes profile and serum lipid profile.**<br>**Histological analysis**<br>**Western blot and RT-PCR for key proteins and enzymes.**<br>**Activation of AMPK pathway**

Supplementary Table S1 – A detailed overview of Berberine and its effects in preclinical studies.

		(3) low-dose DMB group **(20 mg/kg/d,L**) (4) high-dose DMB group (**40 mg/kg/d, H)** (4 weeks) N=8 (1) control group (2) **20 mg/kg/d daily** (4 weeks) In vitro: Different concentration 0-20 μM (24 h)			
Zhao J. et al. (2018) [101]	Sprague-Dawley Rats<br><br>In vivo	BBR 150mg/kg or 250 mg/kg Metformin 150, g/kg or 250 mg/kg (12 weeks) 1. HFD (HFD group)	60 (12/48)	Met and BBR worked by activating AMPK pathway and by inhibiting TLR4/NF-κB-p65 signalling, thus blocking production of proinflammatory cytokines.<br>**Secondary endpoints**:<br>The combination of Metformin and BBR was superior for ameliorating	**Liver enzymes profile and serum lipid profile.**<br>**Histological analysis**<br>**ELISA, Western blot and RT-PCR for key proteins and enzymes.**<br>**Activation of AMPK pathway**

		2. HFD and treatment of 150 mg/kg/d metformin (**HFD + metformin**)<br>3.HFD and treatment of 250 mg/kg/d BBR (**HFD + BBR**)<br>4. HFD and treatment of 150 mg/kg/d metformin plus 250 mg/kg/d BBR (**HFD + metformin + BBR**) for 4 and 8 weeks		steatosis and dyslipidaemia induce by high fat diet.	
Wang Y. et al. (2020) [140]	Macrophages (RAW264.7) and hepatocyte cell lines<br><br>In vitro	BBR (5 μM)<br><br>Cell cultures treated for 1 h with BBR then with palmitic acid (PA) or lipopolysaccha		BBR inhibits free fatty acid (FFA) and LPS-induced inflammatory response in vitro by modulating ER stress and ERK1/2 activation, thus lowering proinflammatory cytokines levels - TNF-α, IL-6, IL-1β and MCP-1<br>**Secondary endpoints:**	**Western blot and RT-PCR key proteins and enzymes.**<br>**ELISA for proinflammatory cytokines levels.**<br>**Western blot**<br>**Endoplasmic reticulum stress response and ERK1/2 pathway.**<br>**PA/LPS inflammatory.**

		ride (LPS) or combination of both for 6 h.		Improvement in stress oxidative response and inflammation.	
Zhang Z. et al. (2016) [132]	Mice C57BL/6J (db/db)<br>HepG2, FAO cell lines. AFML 12<br>In vivo and in vitro	BBR at a dose of 200 mg / kg/ d by gavage (5weeks)<br>In vitro:<br>BBR 5μM		BBR strongly attenuated stress-activated lipogenesis in vivo and in vitro by activating ATF6/SREBP-1c pathway.<br>**Secondary endpoints**:<br>Improvement in liver enzyme profile and serum lipid profile in vivo experiment, as well as improving lipid levels in vitro model.<br>BBR treatment attenuated steatosis (NAS score $p<0.001$) and steatohepatitis both in vitro and in vivo.	**Liver enzymes profile and lipid serum profile**<br>**Western blot and RT-PCR key proteins and enzymes.**<br>**Histological analysis.**<br>**Activation of ATF6/SREBP-1c pathway.**
Yang QH. et al. (2011) [111]	Sprague-Dawley rats<br>In vivo	BBR high-dose (324 mg/kg/d) and low-dose (162 mg/kg/d) by gastrogavage (11 weeks)	40(10/ 10/10/ 10)	Downregulation of mRNA of UCP2 after BBR treatment was associated with modulation of both inflammatory response and lipid metabolism. ($p<0.01$)<br>**Secondary results:**<br>Significant improvement in serum lipid profile after BBR both low and high dose treatment. ($p<0.01$)	**Liver enzymes profile and lipid serum profile.**<br>**Western blot and RT-PCR key proteins and enzymes.**<br>**Histological analysis.**<br>**Immunohistochemistry for protein levels.**<br>**Uncoupling protein-2 (UCP2) mechanism.**

Supplementary Table S1 – A detailed overview of Berberine and its effects in preclinical studies.

		(1) control group<br>(2) model control group<br>(3) the berberine high-dose group (BHD)<br>(4) the berberine low-dose group (BLD		Histological amelioration with no inflammation in BBR treated groups.	
Cossiga V. et al. (2021) [90]	C57BL/6 mice<br>In vivo	Plant extract (140 mg/kg):<br>1.BBR- 87.84 mg<br>2. Tocotrienol – 5.27 mg<br>3.Coffee extract – 5.28 mg<br>(24 weeks)<br><br>(1) standard diet<br>(2) HFD diet<br>(3) HFD + plant extracts (E)	24 (8/8/8)	The plant extracts were able to modulate the microbiota promoting the establishment of specific bacteria, therefore partially correcting dysbiosis induced by high fat diet (HFD)<br>**Secondary endpoints**:<br>Significant improvement in liver enzyme profile ALT (81.3 U/L vs 38.7 U/L, $p < 0.038$, HFD vs HFD+E).<br>Rebalancing glucose metabolism.<br>Minimal (<5%) or absent liver steatosis on histological analysis.	**Liver enzyme profile and serum lipid profile.**<br>**Histological analysis.**<br>**RT-PCR for key enzyme and protein.**<br>**Genomic DNA analysis for gut microbiota.**

Supplementary Table S1 – A detailed overview of Berberine and its effects in preclinical studies.

Dai Y. et al. (2022) [85]	Sprague Dawley rats<br>In vivo	BBR and Evodiamine (E):<br>(1) high dose – 72 mg/kg BBR + 16 mg/kg E<br>(2) medium dose – 36 mg/kg BBR + 8 mg/kg E<br>(3) low dose – 8 mg/kg + 4 mg/kg E<br>(14 weeks)<br><br>(1) control group<br>(2) three groups -high dose BBR+E<br>(3) Medium dose BBR +E<br>(4) low dose BBR + E	60 (10/10 /10/10 /10/10 /10/10)	BE treatment had a positive impact on the recovery of intestinal integrity and improvement of intestinal barrier in HFD-fed mice. Therefor thus by regulating gut microbiota BE exert and anti-NAFLD potency<br>**Secondary endpoints**:<br>BE exerted a protective role by improving liver enzyme levels and serum lipid profile.<br>The anti-NAFLD effects of BE treatment was related to the inhibition of liver inflammation and oxidative stress. – lower levels of proinflammatory cytokines (TNF-a, IL-1b, and IL-6, P<0.001 or p<0.05) and higher levels of anti-inflammatory ones (IL-10, IL-4, P<0.001)	**Liver enzyme profile and serum lipid profile.**<br>**Histological analysis**<br>**qRT-PCR for key enzyme and protein.**<br>**Genomic DNA analysis for gut microbiota**<br>**ELISA for inflammatory cytokines levels.**
Yang S. et al. (2022) [121]	Mice C57BL/6J<br>HepG2 cell cultures	BBR - 40mg/kg<br>BRB – 10/20/40 mg/kg.	70(10/ 10/10 /10/1	BRB acts by enhancing lipolysis mechanism and by reducing lipogenic ones both in vivo and in vitro.	**Liver enzyme profile and serum lipid profile.**<br>**Histological analysis**

Supplementary Table S1 – A detailed overview of Berberine and its effects in preclinical studies.

	In vivo and vitro	MET- 40mg/kg orally once a day<br>(1) control group<br>(2) HFD group<br>(3) MET group<br>(3) BBR group<br>(4) low dose BRB<br>(5) middle dose BRB<br>(6) high dose BRB<br>Cell cultures<br>FEN – fenofibrate – positive control 5μM<br>BRB – 1.25/2.5/5μM.	0/10/10)	**Secondary outcomes**:<br>NAFLD activity score was significatively improved by medium and high dose of BRB, as well as BBR treatment compared to HFD group. ($p<0.01$).<br>Rebalancing the intestinal dysbiosis produced by NAFLD proved to ameliorate lipid and glucose homeostasis both in vivo and in vitro.	**Western blot and RT-PCR for key enzyme and proteins.**<br>**Genomic DNA analysis for gut microbiota.**<br>**Expression of proteins involved in hepatic lipolysis and lipogenesis.**<br>**NAS score.**
Shu X. et al. (2021) [87]	Mice C57BL/6J<br>In vivo	BBR<br>100 mg/kg/d oral gavage	16(8/8)	By modulating gut microbiota, BBR upregulated intestinal FXR expression, thus proving that BBR might ameliorate NAFLD via FXR/FGF-15 pathway.	**RT-PCR and Western blot to detect FXR pathway and FGF-15 levels.**<br>**Liver enzyme profile and lipid profile.**<br>**Genomic DNA analysis for gut microbiota.**

Supplementary Table S1 – A detailed overview of Berberine and its effects in preclinical studies.

		DSS 1% dextran sulphate (16 weeks)<br><br>(1) NASH group (HFD – DSS diet)<br>(2) BBR treated group		**Secondary endpoints**:<br>Liver enzymes profile was significantly ameliorated by BBR treatment between model group and treated group. ($p<0.05$ for ALT and $p<0.001$ for AST).<br>Histological findings showed that liver fat content was decreased by 30% after BBR treatment.	
Chen D. et al. (2023) [84]	Sprague-Dawley rats<br>In vivo	MET 200 mg/kg/day intragastric.<br>BBR 200 mg/kg/day intragastric.<br>(16 weeks)<br>(1) control<br>(2) NASH model<br>(3) MET<br>(4) BER	40 (10/10/10/10)	Histological abnormalities of the liver and NAS score in the control group were significantly ameliorated after BBR and Metformin treatment. ($p< 0.001$).<br>**Secondary endpoints**:<br>Significant improvement concerning liver enzyme profile between model and MET/ BBR groups. ($p<0.001$).<br>Restored intestinal barrier after treatment with BBR/MET ($p<0.001$), thus exerting anti-inflammatory effects by targeting bacterial translocation.	**Liver enzymes profile and lipid profile.**<br>**Hepatic lipid content.**<br>**Liver tissue.**<br>**Liver and spleen microbiota cultures.**<br>**Genomic DNA analysis for gut microbiota.**<br>**RT-PCR for key enzymes and proteins.**
Cao Y. et al. (2016) [106]	Mice BALB/c	BBR (200 mg/kg/d) suspended in	30 (11/10/9)	The NAS score was more than two times lower in the treated group than in the model group. (2.73 vs 5.60).	**Liver enzyme profile and lipid profile**<br>**Histological analysis.**<br>**RT-PCR for key enzymes and protein**

Supplementary Table S1 – A detailed overview of Berberine and its effects in preclinical studies.

	In vivo	distilled water and orally administered. (13 weeks)<br><br>(1) control group<br>(2) model group<br>(3) treatment group		**Secondary endpoints**:<br>Improvements in liver enzyme profile and lipid profile were acquired after BBR treatment.	
Guo T. et al. (2016) [135]	MICE C57BL/6J<br>H4IIE cells (rat hepatoma cells)<br><br>In vivo and in vitro.	BBR (100 mg/kg body weight/day)<br>4 weeks<br><br>BBR (25 μM) – cell culture		BBR treatment modulates the stress oxidative response, as shown by decreased proinflammatory cytokine levels (IL-1β, IL-6, and/or TNFα) in the liver, adipose tissue, and culture cells.<br>**Secondary endpoints**:<br>BBR treatment was associated with rebalancing glucose homeostasis in animal model experiments. ($p<0.05$)<br>BBR did not influence visceral fat mass and adipose tissue.	**Histological analysis.**<br>**Western blot and RT-PCR for key enzymes and proteins.**
Li H. et al. (2022) [122]	Mice C57BL/6J<br>HepG2 cells	Combination of Bicyclol and	50 (ten group	The combination of BBR and bicyclol not only improved lipid metabolism but also ameliorated gut microbiota	**Liver enzyme profile and lipid profile**<br>**Histological analysis.**

		BBR (from 50 mg/kg/day to 200 mg/kg/day) by gavage (12 weeks)	s of 5 mice each)	changes, thus exerting hepatoprotective effects.<br>**Secondary endpoints**:<br>Significant improvement in liver enzyme profile – lower AST and ALT levels were obtained when a combination of BBR (50 mg/kg) was used with Bicyclol (200 mg/kg).	**Western blot and RT-PCR for key enzymes and protein.**<br>**Genomic DNA analysis for gut microbiota.**<br>**NAS score criteria.**
Rafiei H. et al. (2019) [143]	HepG2 cell cultures In vitro	10μM polyphenol, including BBR		BBR upregulated the expression of SIRT1, thus activating the AMPK/SIRT1 pathway. By doing so, mitochondrial activity was enhanced, and different proteins involved in lipid metabolism were phosphorylated, resulting in an anti-steatotic effect.<br>**Secondary endpoints**:<br>BBR treatment and other polyphenols ameliorated steatosis in HepG2 cells.	**Fluorescence imaging**<br>**Western blot and RT-PCR for key enzymes and proteins.**<br>**Mitochondrial and AMPK/SIRT1 pathway activity measurements.**
Zhao L. et al. (2017) [103]	Sprague–Dawley rats In vivo	**BBR** (150 mg/kg body weight/day by gavage. (16 weeks)	18 (6/6/6)	A significant decrease in hepatic lipogenesis ($p<0.05$) and a better control of glucose metabolism were obtained after BBR treatment.<br>**Secondary endpoints**:<br>Histological changes observed in the NAFLD model group were noticeably improved after BBR treatment.	**Liver enzyme profile and serum lipid profile.**<br>**Histological analysis.**

Supplementary Table S1 – A detailed overview of Berberine and its effects in preclinical studies.

		(1) control group<br>(2) NAFLD group<br>(3) BBR treated group		The serum lipid profile, particularly triglyceride levels and total cholesterol, was slightly improved in the BBR-treated group compared to the control group. (TG: 0.70 vs 0.72, TC: 1.55 vs 1.66, LDL: 0.25 vs 0.24)	
Rafiei H. et al. (2023) [138]	Cell cultures HepG2, LX-2 stellate cells, differentiated THP-1 cells.<br>In vitro	BBR 5μM		New perspectives were opened by creating a new triculture model that can mimic NAFL/NASH features for future preclinical studies. In this model, BBR ameliorated oxidative stress response ($p \leq 0.005$) and modulated lipid metabolism.<br>**Secondary endpoint**:<br>A protective effect against lipid accumulation and oxidative stress response was obtained after BBR treatment – lower levels of inflammatory cytokines. ($p \leq 0.006$) and fewer intracellular fat droplets are visible. ($p \leq 0.006$)	**Histological analysis**<br>**ELISA to quantify levels of inflammatory cytokines and chemokines.**<br>**Western blot and RT-PCR for key enzymes and proteins/**<br>**Reactive oxygen species activity.**
Feng WW. et al. (2018) [102]	Sprague-Dawley rats	BBR - 50, 100 mg/kg<br>Curcumin (CUR) - 50, 100 mg/kg	74 (12/62)	The combination of BBR and curcumin proved to significantly downregulate ($p<0.01$) genes related to inflammation compared with the lovastatin treatment group and curcumin or BBR alone groups - SREBP-1c, pERK, TNF-α, and pJNK.	**Liver enzymes profile and serum lipid profile.**<br>**Histological analysis.**<br>**RT-PCR and Western blot for key enzymes and proteins. - protein levels related to inflammation. (SREBP-1c, pERK, TNF-α, and pJNK)**

Supplementary Table S1 – A detailed overview of Berberine and its effects in preclinical studies.

		Lovastatin (LOV) – 100mg/kg intragastrical (20 weeks)<br><br>(A) normal diet n=12<br>(B) high fat diet n=64:<br>(1) Control group<br>(2) High fat group<br>(3) BBR (100mg)<br>(4) Curcumin (100mg)<br>(5) BBR+ Curcumin (50+50 mg)<br>(6) Lovastatin (100)		**Secondary endpoints**:<br>A combination of BBR and curcumin is associated with better control of lipid metabolism and better anti-steatotic effects than lovastatin.	
Wang L. et al. (2021) [91]	Sprague-Dawley rats	BBR 200 mg/kg/day<br><br>PPC 200 mg/kg/day	60 (18/12 /12/12)	BBR significantly reduced *the TLR4/MyD88/NF-κB pathway compared to the control group (p<0.05), thus proving to fight against NAFLD-induced* inflammation.<br>**Secondary endpoints**:	**Liver enzymes profile and serum lipid profile.**<br>**Histological analysis.**<br>**ELISA for inflammatory cytokine levels**

Supplementary Table S1 – A detailed overview of Berberine and its effects in preclinical studies.

		(**Polyene phosphati-dylcholine)** (12 weeks)<br><br>(1) control (normal diet)<br>(2) model (HFD)<br>(3) polyene phosphati-dylcholine HFD+PPC<br>(4) BBR (HFD+BBR) group		Improvement in liver enzyme profile and serum lipid profile<br>Amelioration of histological changes in NAFLD-induced animal model,<br>Anti-inflammatory and hepatoprotective effects.	**RT-PCR and Western blot for key enzymes and proteins.**<br>**NAS score**<br>**Nuclear translocation of NF-κB via the TLR4/MyD88/NF-κB pathway.**
Ye C. et al. (2023) [115]	C57BL/6 mice<br>HepG2 cell culture<br>In vivo and in vitro	BBR (200 mg/kg/d by gavage (12 weeks)<br><br>BBR (5μM) in HepG2 cell culture<br><br>(1) control group<br>(2) model group	27(9/9/9)	BBR significantly alleviates Clock / Bmall1 oxidative stress ($p<0.05$ and $p<0.001$), proving that it may play a protective role by rebalancing circadian rhythm, as well as by modulating lipidic, glucose metabolism, and inflammatory response associated with NAFLD.<br>**Secondary endpoints:**<br>Better control of both lipid and glucose metabolism was obtained after BBR treatment.	**Liver enzyme profile and serum lipide profile.**<br>**ELISA for inflammatory cytokine levels.**<br>**RT-PCR and Western blot for key enzymes and proteins.**<br>**Clock and Bmall1 genes activity.**

Supplementary Table S1 – A detailed overview of Berberine and its effects in preclinical studies.

		(3) BBR group		Histological changes and NAS scores induced in animal model experiments were ameliorated.<br>Lower inflammation was registered after BBR treatment both in vivo and in vitro.	
Yuan X. et al. (2015) [137]	Sprague-Dawley rats<br>Huh7 Human hepatic cell line	BBR 200 mg/kg/day (24 weeks)<br><br>(1) Control group<br>(2) HFD group<br>(3BBR group	24 (8/8/8)	Numerous IncRNAs and mRNAs associated with NAFLD were downregulated after BBR treatment.<br>**Secondary endpoints**:<br>Significant improvement regarding serum lipide profile. ($p<0.05$)<br>Liver histology was ameliorated by BBR treatment.	**Serum lipide profile.**<br>**Histological analysis.**<br>**Gene analysis (IncRNAs and mRNAs)**<br>**RT-PCR for key enzymes and proteins.**
Chen Y. et al. (2021) [120]	C57BL/6 J mice<br>Caco-2 human colon cancer cell line<br>LO2 human normal liver cell line<br><br>In vivo and in vitro	45 mg/kg BER and 15 mg/kg CUR – in vivo<br><br>90 µg/mL BER and 30 µg/mL CUR – in vitro	Not mentioned	Dextran-coated bilosomes containing a combination of BBR and curcumin proved to have superior pharmacokinetics properties on changes induced by NAFLD.<br>**Secondary endpoints:**<br>Significant decrease in intracellular lipid accumulation in cell line model – better effect in this form than normal BBR and CUR traditional form.<br>Superior effects than traditional forms concerning serum lipid profile and liver enzyme profile.	**Liver enzymes profile and serum lipid profile.**<br>**Histological analysis.**<br>**Western blot for key enzymes and proteins.**<br>**NF-κB pathway activity assessment.**

Supplementary Table S1 – A detailed overview of Berberine and its effects in preclinical studies.

				Better anti-inflammatory effects than traditional forms.	
Zhao W. et al. (2016) [105]	Sprague-Dawley rats In vivo	BBR (25, 50, 100 mg) Puerarin, (50, 100, 200 mg) baicalin (25, 50, 100 mg)	96(8/8 /8x9 orthogonal experiments)	The combination of puerarin, baicalin, and berberine upregulates the expression of PPAR-γ and Insulin receptor (IR), thus providing a new pathway for NAFLD treatment.<br>**Secondary endpoints**:<br>Improvement in liver enzyme levels in the puerarin group compared to the model group. ($p<0.01$)<br>Improvement in serum lipid profile, especially in BBR and baicalin-treated groups.<br>Histological improvement was seen in the orthogonal experiment groups and rosiglitazone one.	**Liver enzyme profile and serum lipid profile.**<br>**ELISA for detecting proinflammatory cytokine levels. (TNF-α and IL-6)**<br>**RT-PCR and Western blot for key enzymes and proteins.**<br>**Histological analysis.**<br>**PPAR-γ and Insulin receptor (IR) activation**
Wu L. et al. (2019) [76]	C57BL/6J mice In vivo	BBR - 1.5 mg/kg/day (6 weeks)		BBR treatment stimulated 18F-FDG uptake in adipose tissue, thus increasing thermogenesis and energy expenditure. By doing so, BBR proved to modulate both lipidic and glucose metabolism.<br>**Secondary endpoints**:<br>Significant decrease in body weight after BBR treatment. ($p<0.05$)	**Micro 18F-FDG PET/CT**<br>**RT-PCR and Western blot for key enzymes and proteins.**<br>**Liver enzyme profile and serum lipid profile.**<br>**Temperature and metabolic activity measurements.**<br>**Histological analysis**.<br>**Fat adipose tissue analysis**.

Supplementary Table S1 – A detailed overview of Berberine and its effects in preclinical studies.

				Improvement in glucose metabolism after BBR treatment.<br>BBR upregulated a series of genes associated with brown adipose tissue proliferation and thermogenesis by activating the AMPK–PRDM16 axis signaling pathway.	
Shan MY. et al. (2021) [139]	HepG2 cells culture<br>In vitro	BBR (1,5 and 25 μM)		By activating the SIRT1-FoxO1-SREBP2 pathway, BBR modulated lipid metabolism in steatotic-induced cell cultures.<br>**Secondary endpoint:**<br>A significant difference in cholesterol levels ($p<0.01$) was observed in HepG2 cells.<br>BBR treatment managed to prevent intracellular fat droplet accumulation in vitro.<br>.	**RT-PCR and Western blot for key enzymes and proteins.**<br>**Immunofluorescence.**<br>**SIRT1-FoxO1-SREBP2 pathway downregulation**.
Lu Z. et al. (2020) [95]	Wild-type (WT) Wistar rats<br>In vivo	BBR (300 mg/kg/day	40 (16/16 /8)	Regulating the chemerin/CMKLR1 signaling pathway and restoring the Treg/Th17 cell ratio significantly alleviated liver inflammation, thus	**Liver enzyme profile and serum lipide profile.**<br>**Histological analysis.**<br>**NAS score.**

Supplementary Table S1 – A detailed overview of Berberine and its effects in preclinical studies.

		(1) control group (2) HFD group<br>(3) BBR group		exerting a hepatoprotective effect against NAFLD. ($p<0.01$)<br>**Secondary endpoints**:<br>Improvement in liver enzyme profile and lowered pro-inflammatory levels after BBR treatment.<br>Histological amelioration after BBR treatment.	**Elisa for inflammatory cytokines levels.**<br>**RT-PCR and Western blot for key enzymes and proteins.**<br>**Chemerin/CMKLR1 signaling pathway and Treg/TH17 ratio.**
Wang Y. et al.<br>(2021) [86]	Mixed background C57Bl/6J and 129S1/SvlmJ (B6/12) mice<br>In vivo	BBR (50 mg/kg) by gavage<br>(21 weeks)<br>1. normal diet control group (ND)<br>2. NASH group<br>3. NASH + BBR group (n = 10)	30 (10/10 /10)	By modulating transcriptional factors, BBR treatment managed to increase the expression of 184 genes and decrease the expression of more than 770 genes involved mostly in FAS synthesis and the inflammatory response. Therefore, BBR proved to have anti-steatotic and anti-inflammatory effects by activating different molecular pathways.<br>**Secondary endpoints**:<br>Significant improvement in liver enzyme profile after BBR treatment. ($p<0.05$ and $p<0.001$).<br>Better control of glucose metabolism and lower cholesterol levels, but no improvement in triglycerides or very low-density lipoproteins.<br>Significant improvement in liver histology score regarding steatosis after BBR treatment. ($p<0.001$)	**Liver enzymes profile and serum lipid profile.**<br>**RT-PCR and Western blot for key enzymes and proteins.**<br>**Histological analysis.**<br>**Analysis of multiple cellular pathways involved in NAFLD induction and progression.**

Supplementary Table S1 – A detailed overview of Berberine and its effects in preclinical studies.

He H. et al. (2023) [117]	L02 and HepG2 cell lines<br>C57BL/6J mice<br>In vivo and in vitro	8-cetylberberine (CBBR)- 15 or 30 mg/kg orally<br>(24) weeks<br><br>(1) control group<br>(2) NAFLD model group<br>(3) low dose CBBR<br>(4) high dose CBBR<br><br>CBBR - 2.5, 5, and 10 μM for cells culture	32(8/8/8/8)	In vivo transcriptional analysis revealed that CBBR not only managed to modulate different genes involved in lipid accumulation, inflammation, or steatosis but also downregulated the LCN2 pathway, relieving metabolic changes associated with NAFLD.<br>**Secondary endpoints**:<br>Improvement in serum lipid profile after CBBR treatment in animal model experiment and cell culture.<br>Lower levels of inflammatory cytokines were detected in mice treated with CBBR.	**Liver enzymes profile and serum lipid profile.**<br>**RT-PRC and Western blot for key enzymes and profile.**<br>**Histological analysis.**<br>**Lipocalin-2 (LCN2) gene analysis.**
Yang J. et al. (2017) [104]	C57BL/6J Apolipoprotein E-deficient (ApoE-/-)<br>In vivo	BBR<br>(1) normal diet<br>(2) high-fat high-cholesterol diet (HFHC group)<br>3. HFHC diet supplemented	26(8/8/10)	By activating the CXCR4)/CXCL12 signaling pathway, BBR restored balance among various pro-inflammatory and anti-inflammatory cytokines, proving to have hepatoprotective effects.<br>**Secondary endpoints:**	**Liver enzymes profile and serum lipid profile.**<br>**RT-PRC and Western blot for key enzymes and profile.**<br>**Histological analysis**<br>**C-X-C chemokine receptor type 4 (CXCR4)/CXCL12 signaling pathway**.

Supplementary Table S1 – A detailed overview of Berberine and its effects in preclinical studies.

		with BBR (BBR group		Significant improvement in liver enzyme profile – lower levels of ALT and AST ($p<0.05$)<br>Amelioration of histological disturbances induced by steatosis – lower NAS score ($p<0.05$)	
Luo Y. et al. (2019) [99]	C57BL/6J mice<br>In vivo	BBR 250mg/kg by gavage (12 weeks)<br><br>1. Normal diet<br>2. High fat, high cholesterol diet<br>3. High fat, high cholesterol + BBR	30 (10/10 /10)	By inhibiting the p38MAPK/ERK-COX2 pathway, which is associated with inflammation and angiogenesis, BBR treatment might exert an anti-tumor effect and prevent hepatocellular carcinoma development associated with NAFLD.<br>**Secondary endpoints**:<br>Lower levels of proinflammatory cytokines were registered after BBR treatment.<br>Improvement in liver enzyme profile and lipid serum profile.	**Liver enzymes profile and serum lipid profile.**<br>**RT-PRC and Western blot for key enzymes and profile.**<br>**Histological analysis.**<br>**p38MAPK/ERK-COX2 pathway. analysis**
Ma X. et al. (2023) [114]	HepG2 cells culture<br>BALB/c mice<br>In vivo and in vitro	**BSS**=solid precipitate of BBR and Silybin (100 mg/kg BBR and 120 mg/kg silybin) intragastrically	25(5/5 /5/5/5	Superior bioavailability and effects mediated through a more robust activation of fatty acid transporter CD36 in animal models were obtained using a solid BBR and Silybin precipitate.<br>**Secondary endpoints**:<br>Equivocal results regarding (1H NMR) analysis.	**Hydrogen-1 nuclear magnetic resonance (1H NMR) analysis**<br>**Liver enzymes profile and serum lipid profile.**<br>**RT-PRC and Western blot for key enzymes and profile.**<br>**Histological analysis.**

Supplementary Table S1 – A detailed overview of Berberine and its effects in preclinical studies.

		**BSP=**physical mixture of BBR and Silybin		Both in vivo and in vitro BSS proved to modulate lipid metabolism significantly.	
Li CH. et al. (2018)	HepG2 and MIHA cell culture In vitro	BBR -10 μM and 20 μM		By upregulating the expression of miR-373, BBR activated a series of molecular reactions, which eventually ended with the inactivation of the AKT-S6 kinase pathway. Therefore, BBR alleviated liver steatosis, and insulin resistance mediated through SK6.	**RT-PCR and Western blot for key enzymes and proteins.**<br>**Genome-wide analysis.**<br>**AKT-S6 kinase pathway analysis.**<br>.
Lu Z. et al (2020) [95]	Sprague Dawley rats	BBR - 300 mg/kg intragastric (12 weeks)<br><br>(1) control group<br>(2) high-fat group<br>(3) BBR treated group	24(8/8 /8)	The adipocyte macrophage-derived Angptl2 signaling pathway activation by BBR was associated with significant downregulation of pro-inflammatory proteins.<br>**Secondary endpoints:**<br>Improvement in liver enzyme profile and serum lipid profile after BBR treatment.<br>Lower levels of cytokines show significant anti-inflammatory effects. (CCL2 and TNF-α, p<0.05)	**Liver enzymes profile and serum lipid profile.**<br>**RT-PRC and Western blot for key enzymes and profile.**<br>**Histological analysis**<br>**ELISA for detecting cytokine levels.**<br>**Adipocyte macrophage-derived Angptl2 signaling pathway analysis.**
Deng Y. et al. (2019) [100]	Sprague-Dawley rats	BBR (100 mg/kg b.w) daily (8 weeks)	24(8/8 /8)	BBR treatment was associated with decreased levels of genes involved in inflammatory response. Moreover, by activating the Nrf2/ARE signaling pathway, BBR exerted anti-	**Liver enzymes profile and serum lipide profile.**<br>**RT-PCR and Western blot for key enzymes and proteins.**<br>**Histological analysis.**

		(1) control group<br>(2) HFD group<br>(3) BBR group		inflammatory effects by modulating different key genes associated with the oxidative stress response.<br>**Secondary endpoints:**<br>Liver-induced steatosis was significantly ameliorated by BBR treatment. (p<0.01)<br>Significant improvement in serum lipid profile. (p<0.05)<br>Significant decrease in body weight after BBR treatment. (p<0.01)	**Nrf2/ARE signaling pathway and genes involved in inflammatory response.**
Mehrdoost S. et al. (2021) [88]	Sprague–Dawley rats	BBR - of 150 mg/kg<br><br>Sitagliptin - 10 mg/ kg<br><br>Berberine 75 mg/kg and Sitagliptin 5 mg/kg<br>(6 weeks)<br><br>(1) control group<br>(2) model group<br>(3) BBR group	40(8/8/8/8/8)	The combination of BBR/Sitagliptin significantly upregulated the expression of adiponectin receptor 2 (AdipoR2) – p<0.01. Moreover, co-administration of both substances led to a significant downregulation of ERK levels - a mitogen-activated protein kinase (MAPK) involved in proinflammatory response, thus proving to reduce inflammation associated with NAFLD. (p<0.001)<br>**Secondary endpoints:**<br>Significant improvement in body weight after treatment with a combination of BBR and Sitagliptin. (p<0.001)	**Liver enzyme profile and serum lipid profile.**<br>**Histological analysis.**<br>**RT-PCR and Western blot for key enzymes and proteins.**<br>**Adiponectin receptor 2 (AdipoR2) levels and mitogen-activated protein kinase (MAPK) ERK expression and their influence on inflammation and progression of NAFLD.**

		(4) Sitagliptin group<br>(5)BBR/Sitagliptin group		Improvement in liver enzyme profile and serum lipide profile in all three groups.<br>No fatty degeneration was detected in the combination drug-treated group.	
Zhou LM. et al. (2023) [118]	C57BL/6 J wild-type (WT) mice and<br>HepG2 cell lines<br>In vivo and in vitro	Epiberberine (EPI) – 50,100 and 200 mg/kg (45 days)<br>(1) control group<br>(2) methionine-choline-deficient diet<br>(3) low dose EPI<br>(4) medium dose EPI<br>(5) high dose EPI	25 (5/5/5/5/5)	Using animal and cell experimental models, EPI improved steatotic-induced liver lesions by suppressing the SREBP1/FASN pathway, thus modulating lipid synthesis and accumulation in the liver.<br>**Secondary endpoints:**<br>No changes in weight after EPI treatment.<br>EPI ameliorated inflammation by decreasing levels of cytokines, especially CD68.<br>Rebalancing gut dysbiosis, thus increasing beneficial gut flora and ameliorating inflammatory response.	**Liver enzyme profile and serum lipid profile.**<br>**RT-PCR and Western blot for key enzymes and proteins.**<br>**Histological analysis.**<br>**Immunofluorescence for detecting inflammatory molecules.**<br>**SREBP1/FASN pathway analysis.**
Xing LJ. Et al. (2011) [112]	Wistar rats<br>In vivo	**BBR** 187.5 mg/kg/day<br>**Pioglitazone** 10.0 mg/kg<br>**Saline** 3 ml/kg<br>(4 weeks)	40 (10/10/10/10)	RT-PCR was used to detect ISR-2 mRNA expression. BBR treatment was associated with significant upregulation of ISR-2 levels (p<0.01), thus modulating insulin resistance and	**Liver enzyme profile and serum lipid profile.**<br>**RT-PCR and Western blot for key enzymes and proteins.**<br>**Histological analysis.**

Supplementary Table S1 – A detailed overview of Berberine and its effects in preclinical studies.

		(1) control group<br>(2) HFD + BBR<br>(3) HFD + Pioglitazone<br>(4) HFD + saline		ameliorating metabolic changes induced by NAFD.<br>**Secondary endpoints:**<br>No changes in serum lipid profile were registered after BBR treatment.<br>Histological changes amelioration after BBR treatment.	**Insulin receptor substrate-2 (ISR-2) levels analysis**.
Wang P. et al. (2023) [119]	HepG2 and AML12 cells line<br>C57BL/6J mice<br>In vivo and in vitro	BBR 10 or 20 μM for cell lines<br>BBR<br>200 mg/kg/d by gavage<br>(4 weeks)		BBR ameliorated changes induced by NAFLD through the upregulation of SRT1 and CPT1A. Moreover, by enhancing SIRT1 and CPT1A levels both in vivo and in vitro, BBR increased fatty acid oxidation and decreased intracellular lipid accumulation.<br>**Secondary endpoints:**<br>BBR treatment was associated with amelioration of glucose metabolism. (6.4 vs 5.3, P<0.01).<br>Improvement in liver enzyme profile – (lower AST and ALT levels, p<0.001) as well as improvement in serum lipid profile (lower levels of TG – p<0.004, TC and LDL-c, p<0.001)	**Liver enzymes profile and serum lipide profile.**<br>**Histological analysis.**<br>**RT-PCR and Western blot for key enzymes and proteins.**<br>**SIRT1 and CPT1A levels.**
Xu X. et al. (2019) [98]	C57BL/6J mice<br>In vivo	BBR<br>300 mg/kg/d<br>(12 weeks)	40(10/10/10/10)	Mitochondrial β-oxidation was enhanced via the SIRT3 pathway after	**Serum lipid profile.**<br>**Histological analysis.**

Supplementary Table S1 – A detailed overview of Berberine and its effects in preclinical studies.

		(1) control group<br>(2) control group + BBR<br>(3) HFD<br>(4) HFD+BBR		BBR treatment, proving that BBR has anti-steatotic properties.<br>**Secondary endpoints:**<br>Better control of lipid and glucose metabolism after BBR treatment.<br>Histological amelioration after BBR treatment	**RT-PCR and Western blot for key enzymes and proteins.**<br>**SIRT3 pathway and its effects on lipid metabolism.**<br>**Mitochondrial β-oxidation levels.**
Mai W. et al. (2020) [126]	AML12 cell culture and<br>C57BLKS/J mice<br>In vivo and in vitro	BBR (10 or 20 μM) – cell culture<br>BBR (100 mg/ kg body weight/d,)- by gavage<br>(12 weeks)<br>(1) MCS diet<br>(2) MCD diet<br>(3) BBR group	15(5/5 /5)	BBR significantly inhibited NLRP3 activity in both in vivo and in vitro experiments, proving that it has significant anti-inflammatory properties.<br>**Secondary endpoints**:<br>Significant histological amelioration in model animal experiment.<br>Better control of the oxidative stress response both in vivo and in vitro—lower levels of TNF-α, NLRP3, and caspase-1.	**Histological analysis.**<br>**RT-PCR and Western blot for key enzymes and proteins.**<br>**Liver enzyme profile.**<br>**Fluorescence for reactive oxygen species.**<br>**Caspase-1 and Nod-like receptor family pyrin domain containing 3 (NLRP3) inflammasome activities analysis.**
He Q. et al. (2016) [134]	LO2 human cell line<br>C57BL/6J mice<br>In vivo and in vitro	BBR (50 mg/kg/day)<br>CQ - chloroquine 25 mg/kg/day	50(10/ 10/10/ 10/10)	BBR had a significant impact both in vivo and in vitro on lipid metabolism, as shown by inducing liver autophagy and preventing lipid accumulation via ERK-dependent mTOR pathway.<br>**Secondary endpoints:**	**Immunohistochemistry and histological analysis.**<br>**RT-PCR and Western blot 1 for key enzymes and proteins.**<br>**Liver and serum lipid profile.**<br>**Autophagy induced by activated ERK-dependent mTOR pathway.**

		BBR 50 mg/kg/day) + CQ (5 mg/kg/day (12 weeks)<br><br>BBR (0.2, 1, 5μM) – cell line<br><br>(1) control group<br>(2) HFD<br>(3) BBR group<br>(4) CQ group<br>(5) Co-administration group		Significant improvement in TC and TG serum levels after BBR treatment. ($p<0.05$)<br>Reduced body weight ($p<0.01$) and better control of glucose metabolism.<br>Noticeably decrease in intracellular lipid accumulation both in vivo and in vitro.	
Sun Y. et al. (2017) [130]	C57BL/6 mice<br>Sprague Dawley rats<br>Human hepatoma cell lines Huh7 and HepG2<br>In vivo and in vitro	BBR 300 mg/kg/d = mice<br><br>BBR 200 mg/kg/d= rats (24 weeks)		BBR treatment was associated with downregulation of the Nrf2/HO-1 pathway expression and decreased reactive oxygen species, thus exerting anti-inflammatory effects both in vivo and in vitro.<br>**Secondary endpoints:**<br>BBR particularly actioned on I and III mitochondrial respiratory chains.	**RT-PCR and Western blot for key enzymes and proteins.**<br>**Fluorescence analysis of stress oxidative response.**<br>**Liver triglyceride levels.**<br>**Mn-SOD activity measurement and mitochondrial respiratory chain activity.**<br>**Nrf2/HO-1 pathway activity.**

Supplementary Table S1 – A detailed overview of Berberine and its effects in preclinical studies.

Rafiei H. et al. (2017) [144]	HepG2 cell line<br>In vitro	Polyphenols including BBR (1, 5, 10 μM)		BBR was the strongest polyphenol that inhibited the generation of reactive oxygen species.<br>**Secondary endpoints:**<br>BBR did not affect mitochondrial manganese superoxide dismutase (Mn-SOD).<br>Polyphenols modulated mitochondrial respiratory complex subunits, thus providing critical anti-inflammatory properties.	**RT-PCR for key enzymes and proteins.**<br>**Fluorescence analysis for reactive oxygen species analysis.**<br>**Effects of different polyphenols against stress oxidative response.**
Liang H. et al. (2018) [129]	C57BL/6J mice<br>Cell cultures<br>In vivo and in vitro	BBR (200 mg/kg/d) in vivo experiment, intragastric administration<br><br>BBR 20 μM – in vitro experiment		BBR abolished phosphorylation, thus reducing ABCA1 levels. By doing so, BBR, via the protein kinase C δ pathway, reduced lipid levels and modulated lipid metabolism both in vivo and in vitro, proving that it has anti-steatotic effects.<br>**Secondary endpoints:**<br>Significant improvement of liver enzyme levels – lower levels of AST and ALT after BBR treatment compared to the model group. ($p<0.05$).<br>There was a significant decrease of TG liver levels in the treated group in the	**Intracellular and liver lipid profile.**<br>**Liver enzyme profile.**<br>**RT-PCR and Western blot for key enzymes and proteins.**<br>**Histological analysis.**<br>**ATP-binding cassette transporter A1 (ABCA1) and protein kinase C δ pathway activities.**

Supplementary Table S1 – A detailed overview of Berberine and its effects in preclinical studies.

				animal experiment (p<0.01), but no significant changes in serum ones.<br>BBR prevented intracellular lipid accumulation in vitro. (p<0.05)<br>Histological amelioration of liver of HDF induced alteration after BBR treatment in animal experiment.	
Zhang Y. et al (2015) [136]	Wistar rats<br><br>Cell cultures<br><br>In vivo and in vitro	BBR 200 mg/kg/d by gavage<br>(16 weeks)<br><br>(1) control group<br>(2) NAFLD model group<br>(3) BBR group	15 (5/5/5)	BBR ameliorated both lipidic and glucose metabolism by upregulating the expression of L-PK (p<0.001), as well as by promoting acetylation of histones (resi dues H3K9, H3K18, H4K8, H4K12) involved in NAFLD pathophysiology.<br>**Secondary endpoints:**<br>Significant weight loss on an animal model. Treated with BBR compared to the HFD group. (p<0.05)<br>There was a significant decrease in visceral fat in the treated group compared to model one. (74.7g vs 42.7g, p<0,05).<br>Histological amelioration of changes induced by HFD after BBR treatment.	**Histological analysis.**<br>**RT-PCR for key enzymes and proteins.**<br>**L-pyruvate kinase (LK) activity assessment.**
Teodoro JS. et al., (2013) [110]	Sprague–Dawley-rats	BBR 100 mg/kg/d<br>(16 weeks)		BBR completely restored mitochondrial activity, thus proving to have hepatoprotective and anti-	**Liver enzyme profile and serum lipid profile.**<br>**RT-PCR and Western blot for key enzymes and proteins.**

Supplementary Table S1 – A detailed overview of Berberine and its effects in preclinical studies.

		(1) control group<br>(2) model group<br>(3) BBR group		inflammatory effects via upregulation of SIRT3 ($p<0.05$).<br>**Secondary endpoints:**<br>BBR treatment was associated with a significant decrease in body weight ($p<0.05$)<br>BBR treatment recovered insulin sensitivity. ($p<0.05$)<br>There is no significant improvement regarding the liver enzyme profile.<br>Lower levels of ROS were generated under BBR treatment. ($p<0.05$)	**Mitochondrial activity and reactive oxygen species levels.**<br>**SIRT3 activity level measurements.**
Ragab SM. et al. (2015) [108]	Wistar rats	BBR -50 mg/kg b.w<br><br>Quercetin (Q) -50 mg/kg b.w.<br><br>O-coumaric acid (CA) -75 mg/kg b.w<br><br>(6 weeks)<br>(1) control group<br>(2) HFD model group<br>(3) BBR group	50(10/10/10/10/10)	Significant upregulation of PPARγ expression was achieved after BBR treatment ($p<0.01$), thus modulating lipid metabolism.<br>**Secondary endpoints:**<br>No significant decrease in hepatic lipid content was achieved.<br>A significant improvement in serum lipid profile was obtained after BBR treatment. ($p<0.001$)	**Liver enzymes and serum lipid profile.**<br>**Measurements of liver enzymes involved in lipid metabolism activities.**<br>**Histological analysis.**<br>**Upregulation of peroxisome proliferator-activated receptor γ (PPARγ) in adipose tissue and the liver**

Supplementary Table S1 – A detailed overview of Berberine and its effects in preclinical studies.

		(4) Q Group<br>(5) CA group			
Zhang Y. et al. (2021) [124]	C57BL/6 mice<br>L02 cell culture<br>In vivo and in vitro	Demethylenetetrahydroberberine (DMTHB) 50 or 150 mg/kg - in vivo<br>(30 days)<br><br>DMTHB 10 and 15 μM- in vitro<br><br>(1) control diets<br>(2) MCD diets<br>(3) MCD diets+150 mg/kg silymarin (ig)<br>(4) MCD diets+50 mg/kg DMTHB (ig),<br>95) MCD diets+150 mg/kg	40(8/8/8/8/8)	DMTHB activates the NRLP3 pathway and decreases the ER stress response, which proves it to have superior anti-inflammatory and hepatoprotective properties than BBR or DMB.<br>**Secondary endpoints:**<br>Significant improvement in liver enzyme profile was observed in the DMTHB-treated group compared to the model group. ($p<0.0001$)<br>Histological amelioration after DMTHB treatment.<br>Significant improvement regarding serum lipid profile. ($p<0.01$)<br>Anti-inflammatory effects exerted by DMTHB include lower levels of proinflammatory cytokines. (TNF-α, IL-1β and IL-6)	**Liver enzyme profile and serum lipid profile.**<br>**Histological analysis.**<br>**RT-PCR and Western blot for key enzymes and proteins.**<br>**Pro-inflammatory cytokines levels analysis.**<br>**NRLP3 pathway and stress oxidative response.**<br><br>.
